# Tongue-Tie Assessment and Division: A Time-Critical Intervention to Optimise Breastfeeding

**Published:** 2015-01-10

**Authors:** Jack Donati-Bourne, Zainab Batool, Charles Hendrickse, Douglas Bowley

**Affiliations:** 1 University Hospitals North Midlands NHS Trust, UK; 2 Heart of England NHS Foundation Trust, UK

**Keywords:** Frenulotomy, Tongue-tie, Breast-feeding

## Abstract

Objectives: Recent reports have highlighted the benefits of surgical division of tongue-tie (frenulotomy) in infants with breastfeeding difficulties. There is no clear consensus defining the appropriate age for this procedure to be undertaken in selected infants.

We aimed to evaluate the impact of delays in time between referral and frenulotomy in relation to maternal abandonment of breastfeeding.

Materials and Methods: This was a prospective cohort study done in out-patient Neonatal Surgery Department, Birmingham Heartlands Hospital, Birmingham, UK, between April 2013 and July 2013.

All infants, referred to our tongue-tie clinic between April and July 2013, were studied prospectively. Referral time lags were calculated using computer records; details regarding breastfeeding were collected by an independent interviewer completing a questionnaire.

Results: Seventy patients were included. The median infant age at clinic was 28.5 days [range 1-126]. Fifty eight [82%] of mothers had breastfeeding difficulty and their infants were confirmed to have a prominent tongue-tie. By the time of their clinic attendance, breastfeeding had either not been established or abandoned in 21%. Despite difficulty, 61% of mothers persisted breastfeeding and all these mothers consented for frenulotomy. At time of clinic, median age of infants whose mothers had abandoned breastfeeding was 37 days [range 1-80] compared to 27 days [range 1-126] in infants whose mothers had persisted.

Conclusions: We demonstrated a time-critical dimension for frenulotomy: delay beyond 4-weeks from referral to assessment of neonatal tongue-tie is more likely to be associated with abandonment of breastfeeding. Timely assessment and division of tongue-tie in selected infants can therefore play an important role in a birthing unit’s breastfeeding strategy.

## INTRODUCTION

The presence of a neonatal tongue tie and its surgical division (frenulotomy) is an ancient procedure – operative interventions were documented in ancient Greek medicine.[1] Enthusiasm for its practice has been revived in recent times.[2] Despite ongoing scepticism,[3] recent years have seen a profusion of reports highlighting the benefits of frenulotomy in infants with breastfeeding difficulty.[2][4] A tongue-tie assessment and division service was established in our hospital in 2006, with the specific aim of helping struggling mothers to establish and maintain breastfeeding. Referrals to our clinic however have increased to the point where delays of several weeks to consultation can occur. We aimed to evaluate our current practice with the goal of refining our tongue-tie service, as well as to assess the impact of delays in time to assessment in relation to maternal abandonment of breastfeeding.

## MATERIALS AND METHODS

All infant patients, referred for tongue-tie assessment between April 2013 and July 2013, were included in the study. No exclusion criteria were applied. Data were collected by questionnaire, completed by an independent observer who interviewed the infants’ parents/carers immediately after the clinic consultation. Maternal informed consent was obtained prior to interview.

Information was obtained at interview regarding:

- Details of the referring practitioner,
- Feeding method at time of clinic attendance,
- Whether mother had persisted or abandoned breastfeeding,
- Maternal experience on neonatal latch, breast pain and their overall breastfeeding experience, (subjective scoring scale of increasing satisfaction between 1 – 10)
- Whether the tongue tie had been divided at consultation.

Delay between date of referral and clinic appointment was calculated by reviewing scanned electronic records. Patients were followed up by telephone consultation a median of 3.3 weeks [range 1-6] after the clinic appointment. 

Information was obtained regarding:

- Method of feeding post-clinic
- Complications
- Reported maternal alteration in breast pain, latch and overall breastfeeding experience.

Mann-Whitney Test was used for statistical analysis to compare median age of infants whose mothers had abandoned breastfeeding before their appointment to infants whose mothers persisted in breastfeeding.

This work was considered to be a Service Evaluation Project and, therefore, exempt from ethical approval.

## RESULTS

A total of (n = 70) patients [48 male, 22 female] were included in the study. Median infant age at clinic appointment was 28.5 days [range 1-126]. The sources of the referrals are shown in [Table 1].

**Figure:**
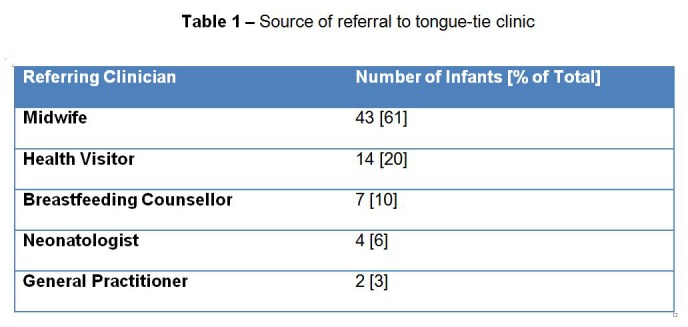
Table 1 – Source of referral to tongue-tie clinic

Median delay from date of referral to date of attendance was 23 days [range 1-41], and only 14/70 [20%] infants had a delay to clinic of 2 weeks or less. 

Inappropriate referrals were noted in 11/70 [16%] infants, where no tongue-tie could be identified [3/70, 4.3%] and/or there had never been any documented breastfeeding difficulty [8/70, 11.7%], and these were excluded from analyses. One older child was booked for elective division under general anaesthetic, and therefore also excluded from analyses.

Fifty eight [82%] mothers had experienced breastfeeding difficulty and their infants were confirmed to have a prominent tongue-tie. In this group however, by the time of their clinic attendance, breastfeeding had been abandoned in 15/58 [26%] infants. Despite difficulty, 43/58 [74%] mothers had persisted in attempts to breastfeed and tongue-tie division [frenulotomy] was offered. 

Median age of infants whose mothers had abandoned breastfeeding before their appointment was 37 days [range 1- 80] compared to 27 days [range 1-126] in infants whose mothers persisted in breastfeeding [p=0.0951, Mann-Whitney Test]. 

Proportion of babies abandoning breast feeding by time of delay to clinic appointment is shown in (Fig.1).

**Figure:**
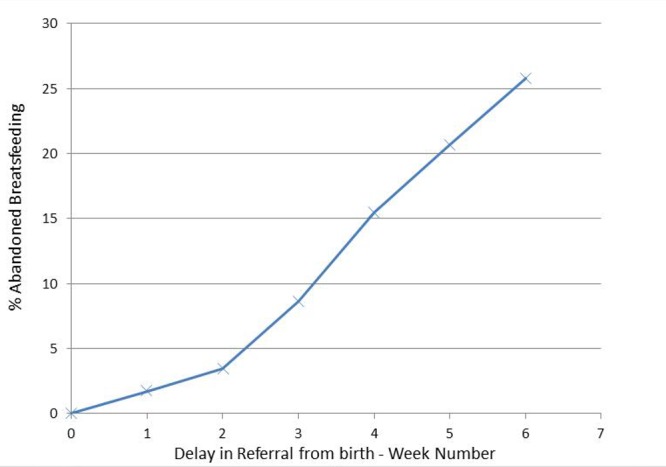
Figure 1: Proportion of babies abandoning breastfeeding by time of delay to clinic appointment.

Mothers whose infants underwent frenulotomy reported by means of subjective scoring scale, significant improvements in pain [70%], latch [75%], and overall breastfeeding experience [80%]. No complications were reported at follow-up telephone consultation.

## DISCUSSION

Tongue-tie division is a safe procedure, and our study reveals significant benefits for maternal breastfeeding, with 75% reporting improvement in latch and 80% in overall breastfeeding experience.

The results of our survey are consistent with reports from early enthusiasts for tongue tie division. In the study of 215 infants by Griffiths, [5] 80% of those who underwent frenulotomy were found to have reported improved feeding at 24 hours by their mothers. Dollberg et al. [6] studied a group of 264 mothers of infants who had undergone frenulotomy for breastfeeding difficulties, by following them up for 6 months via telephone questionnaire, and reported an improvement in breastfeeding in 75% of cases.

Furthermore in the double-blind, randomized controlled trial of tongue-tie division, Berry et al. [7] found that 78% of mothers whose infant had undergone frenulotomy reported immediate improvement in feeding, compared to 47% of those in the no-treatment group who also reported immediate improvement.

In a randomized controlled-trial in Bristol, Emond et al. [8] studied 107 infants allocated at random to an ‘early frenulotomy intervention’ group or a ‘standard care’ comparison group. The median age of infants was 11 days. The standard care group were given the routine breastfeeding support by midwives; however mothers in this group who were still experiencing difficulties after 5 days into the study were given the option of frenulotomy. They reported 85% of mothers requesting frenulotomy either within the 5 day period or immediately after it, due to ongoing breastfeeding difficulties. It is therefore reasonable to suggest that these struggling mothers would have otherwise abandoned breastfeeding within 4 weeks. At 8 weeks follow up, the breastfeeding rate amongst mothers whose infants had not had a frenulotomy was only 12%, compared to the national UK average breastfeeding rate of 47% for infants aged 8 weeks. [9]

Buryk et al. [10] conducted a randomized single-blind controlled trial in Portsmouth, comparing a group of infants taken into clinic for frenulotomy with a ‘sham’ group, where no procedure was performed, all parents blinded to treatment. Mothers were only informed of their study group after the first breastfeed, and were given the option to have a frenulotomy within 2 weeks if their baby had not had it. 96% of mothers further requested frenulotomy, due to ongoing breastfeeding difficulties.

Buryk et al. recommend that the optimal timing for frenulotomy is 2-6 days after birth, to allow for a reasonable attempt at establishing breastfeeding patterns. Earlier division has been shown to result in quicker recovery of feeding patterns, [11] and other recommendations include division for symptomatic babies by age of 2 weeks. [7]

There were limitations to our study. It was not possible to follow-up by telephone all the mothers at the same time interval from consultation date, and this could have led to recall bias. Furthermore, we did not implement any randomization or blinding, as our key aim was to assess the effect of delay in referral, which was calculated at their arrival to clinic. 

Our study adds to the modern literature concerning tongue tie division; in our service, if the interval from referral to assessment extends beyond 4 weeks, the mothers are more likely to have abandoned difficult breastfeeding compared to those who were seen earlier.

## Conclusion

Frenulotomy is a simple, safe procedure, and is associated with significant improvements in maternal reported pain, latch and overall breastfeeding experience in the majority of instances.

The time critical dimension for this intervention is confirmed by our study: delay beyond 4 weeks from referral to assessment of neonatal tongue tie is more likely to be associated with abandonment of breast feeding. 

Departments should organise themselves such that referrals are confined to infants with prominent tongue tie and whose mothers are struggling with breastfeeding; early intervention should be the goal.

## Footnotes

**Source of Support:** None

**Conflict of Interest:** None

